# Methane Concentration Prediction in Anaerobic Codigestion Using Multiple Linear Regression with Integrated Microbial and Operational Data

**DOI:** 10.3390/bioengineering12111133

**Published:** 2025-10-22

**Authors:** Iván Ostos, Iván Ruiz, Diego Cruz, Luz Marina Flórez-Pardo

**Affiliations:** 1 Grupo de Investigación en Ingeniería Electrónica, Industrial, Ambiental, Metrología GIEIAM, Universidad Santiago de Cali, Cali 760036, Colombia; 2 Grupo de Interacciones Microbianas GIM, Universidad de Salamanca, 37008 Salamanca, Spain; diegofagua@usal.es; 3 Grupo de Investigación en Modelado, Análisis y Simulación de Procesos Ambientales e Industriales PAI+, Universidad Autónoma de Occidente, Cali 760030, Colombia

**Keywords:** codigestion, metagenomics, biogas, MLR

## Abstract

Anaerobic codigestion of organic residues is a proven strategy for enhancing methane recovery. However, the complexity of microbial interactions and variability in operational conditions make it difficult to estimate methane concentration in real time, particularly in rural contexts. This study developed a multiple linear regression model to predict methane concentration using operational data and microbial community profiles derived from 16S rRNA gene sequencing. The system involved the codigestion of cassava by-product and pig manure in a two-phase anaerobic reactor. Predictor variables were selected through a hybrid approach combining statistical correlation with microbial functional relevance. The final model, trained on 70% of the dataset, demonstrated satisfactory generalization capability on the other 30 test set, achieving a coefficient of determination (R^2^) of 0.92 and a mean relative error (MRE) of 6.50%. Requiring only a limited set of inputs and minimal computational resources, the model offers a practical and accessible solution for estimating methane levels in decentralized systems. The integration of microbial community data represents a meaningful innovation, improving prediction by capturing biological variation not reflected in operational parameters alone. This approach can support local decision making and contribute to Sustainable Development Goal 7 by promoting reliable and affordable technologies for clean energy generation in rural and resource-constrained settings.

## 1. Introduction

The Valle del Cauca region is one of Colombia’s most active agro-industrial areas, combining high agricultural productivity with unique ecological richness. The territory is sustained by ecosystems that range from coastal plains to montane forests, which support both biological diversity and productive capacity. It ranks as the third-largest producer and consumer of pork in the country, with a reported output of 88,105 tons in 2023, equivalent to 15.6% of national production, and an average pig population exceeding 396,000 animals [1]. This sector generates large volumes of pig manure (PM) that require appropriate handling to prevent environmental and public health risks. Another productive activity with growing regional relevance is cassava cultivation, which covered approximately 564 hectares in 2020, yielding a total of 9888 tons of fresh roots [2]. During starch extraction, each kilogram of cassava generates about 0.2 kg of starch, 0.65 kg of fibrous residue (cassava dregs (CD)), and between five and seven liters of wastewater [3,4]. Based on these ratios, the estimated annual generation of by-products in the region reaches nearly 6427 tons, most of which are not currently valorized.

To address the increasing accumulation of organic residues from pig farming and cassava processing, anaerobic digestion (AD) has been promoted in rural areas of Valle del Cauca as a strategy for energy recovery and waste management. In these settings, one-phase tubular biodigesters are commonly employed due to their affordable construction, ease of installation and minimal infrastructure requirements, making them particularly attractive to smallholder producers [5].

AD is a biologically mediated process capable of metabolizing up to 95% organic matter [6]. It proceeds through four main sequential stages, each driven by specific microbial groups. In the hydrolysis phase, hydrolytic bacteria degrade complex macromolecules such as carbohydrates, proteins and lipids into soluble monomers. During acidogenesis, these compounds are converted by fermentative bacteria into volatile fatty acids (VFAs), alcohols, hydrogen and carbon dioxide. In acetogenesis, acetogenic microorganisms convert these intermediates into acetate, along with additional hydrogen and carbon dioxide. Finally, in methanogenesis, archaea utilize acetate, hydrogen and carbon dioxide to generate methane as the principal end product [7]. While each stage performs a distinct role, the overall efficiency of this multistep pathway depends on the synchronized activity of these microbial groups, where syntrophic cross-feeding and interspecies H_2_/formate transfer channel mediates toward effective substrate valorization and stable methane formation [6,8,9]. Beyond its biological complexity, AD offers important advantages, including the provision of reliable baseload renewable energy that is independent of weather conditions, the achievement of high energy yields per unit area once stabilized, and the generation of multiple energy outputs such as biomethane, hydromethane, electricity, heat, and biohydrogen [7].

For the process to remain stable and efficient, environmental conditions such as pH and temperature must be kept within optimal ranges, typically between 6.5 and 7.5 for pH and 30 to 38 °C under mesophilic conditions [10,11]. In addition, maintaining a C:N ratio between 20:1 and 30:1 is considered ideal for AD, as it ensures sufficient nitrogen for microbial growth without leading to ammonia inhibition or carbon limitation [12,13]. However, most rural systems lack monitoring tools and operate through empirical practices, without clear understanding of internal conditions or microbial dynamics [5,14]. This limitation frequently leads to process imbalance, reduced performance and early system failure.

To overcome the performance limitations of conventional digesters, several strategies have been developed to improve substrate biodegradability and enhance biogas production. Among them, mechanical pre-treatments, codigestion, and multiphase configurations have proven to be particularly effective in increasing system efficiency [15,16,17]. Mechanical pre-treatments have proven effective in enhancing the hydrolysis of lignocellulosic substrates by reducing particle size and fiber crystallinity, thus increasing surface area and enzymatic accessibility [15,18]. Depending on specific conditions, methane production improvements of 16% to 99% have been reported with mechanical treatments [19]. These results highlight the potential of simple mechanical treatments to enhance biodegradability and biogas productivity, especially during the hydrolysis and acidogenesis phases, which are often rate-limited in solid waste digestion.

Codigestion has emerged as a robust strategy to address the nutrient imbalances and low biodegradability often associated with single-substrate digestion. By combining complementary feedstocks, this approach improves the carbon to nitrogen (C:N) ratio, dilutes inhibitors, and stimulates microbial activity, allowing for higher energy yields [16]. For instance, it has been reported that mixtures containing 66% PM, 16% cassava pulp, and 16% bagasse achieve higher methane yields than those with high bagasse content alone, which led to pH imbalances and process failure [20]. Likewise, biogas production efficiency and system stability for food waste and corn straw co-digestion with a hydraulic retention time (HRT) of 25 days have been informed, showing that codigestion notably enhanced the efficiency of the hydrolysis and acidogenesis stages, with the highest anaerobic biodegradability (85.7%) obtained when the food waste content was set at 60% [21]. These improvements are attributed to enhanced microbial synergy and substrate availability, which accelerate volatile solids degradation.

Multiphase AD systems have been developed to address the limitations of single-stage configurations by creating distinct operational environments for each metabolic phase [17]. In two-phase systems, the acidogenic and methanogenic stages are physically separated, which enables more efficient substrate conversion, greater resilience to organic shocks, and better pH control [4,17]. This structural decoupling has led to increases in methane yields, improved volatile solids removal, and significant reductions in HRT without compromising performance [4]. Although three-phase systems further refine process compartmentalization by isolating hydrolysis, acidogenesis, and methanogenesis, they often entail higher operational complexity, energy consumption, and maintenance requirements [22]. These drawbacks have limited their scalability, particularly in low-resource contexts. Consequently, two-phase systems represent a practical balance between performance enhancement and technical feasibility, making them a more accessible alternative for decentralized applications.

Among the strategies developed to improve AD performance, the integration of real-time monitoring systems has become increasingly relevant for enhancing process oversight and operational efficiency [23]. Basic and key variables such as pH, temperature, and methane concentration can be considered to infer the internal state of the reactor and anticipate potential imbalances. The use of cost-effective IoT platforms such as ESP32 microcontrollers coupled with sensors has proven suitable for real-time tracking, achieving deviations below 2% for CH_4_ and 1.7% for pH when compared to laboratory-grade methods [23,24]. Systems incorporating the MQ-4 sensor (200–10,000 ppm CH_4_) and platforms like ThingSpeak facilitate continuous data acquisition, cloud visualization, and automatic alerts, offering a practical solution to reduce manual intervention and increase system reliability [23,25,26].

In parallel, greater attention should be given to the microbial community (MC) involved in AD, as they are rarely considered in routine operation despite being responsible for driving the entire process. Recent studies have highlighted that variations in microbial structure are strongly influenced by substrate type, operational parameters such as temperature and organic loading rates (OLR), and reactor configuration. However, most operational strategies still rely exclusively on physicochemical parameters, overlooking microbial signals often preceding system imbalances [7]. With this in mind, evidence from studies on hunger stress has demonstrated that shifts in microbial communities under adverse conditions provide valuable insights into process behavior and system dynamics, underscoring the importance of integrating microbial data into process understanding to clarify how structural and functional changes within the community influence methane levels [27].

Despite their central role in AD, MLR models have traditionally been developed using operational variables that capture external system conditions, parameters that are directly measurable or predefined during setup, while MC have often been treated as secondary inputs or excluded altogether. For instance, recent studies have used MLR to predict specific methane production from dry AD of the organic fraction of municipal solid waste in pilot-scale plug-flow reactors. Six significant, mostly operational predictors were prioritized (VS, OLR, HRT, C/N ratio, lignin content, and VFA) via Pearson correlation and PCA. Simple regression showed low performance (R^2^ = 0.3), while the full MLR reached R^2^ = 0.91. A reduced model with four uncorrelated variables (VS, OLR, C/N ratio, lignin content) maintained strong accuracy (R^2^ = 0.87) with fewer inputs [28]. Similarly, MLR has been applied to predict VFA concentrations in AD of primary and secondary sludge using operational and physicochemical inputs. The model achieved R^2^ values above 0.85 in several scenarios, offering high interpretability and low computational demand. Although less accurate than leading ensemble methods, MLR remains suitable for applications that require clear interpretation of variable influence [29].

Unlike models based solely on operational parameters, recent full-scale work in thermophilic dry methane systems showed that MC remained stable, with *Methanoculleus* and syntrophic acetate oxidizers dominating throughout the process. This stability enabled the development of an adjusted MLR model which achieved high predictive accuracy (R^2^ = 0.97) and outperformed gradient boosting approaches, highlighting the importance of linking microbial consistency with operational data for reliable large-scale biogas prediction [30].

Building on emerging evidence supporting the integration of microbial data into statistical modeling, this study aims to develop a predictive model for methane concentration based on a set of measurable variables, including VFAs, microbial populations, and operational parameters. It evaluates the potential of MLR to predict methane concentrations in a low-cost, two-phase anaerobic digester treating PM and CD at laboratory scale. This work aligns with Sustainable Development Goal 7 by promoting accessible tools for energy generation from organic waste.

The article is structured into four main sections. The Introduction outlines the context of AD in the Valle del Cauca region, highlighting environmental and operational challenges from agro-industrial organic waste, reviewing strategies to improve biogas systems, and emphasizing the need to integrate microbial data into predictive models. The Materials and methods detail the system setup, monitoring, sequencing, and the MLR approach used for variable selection and model construction. The Results and Discussion sections present the modeling outcomes, identify relevant predictors, and interpret their contribution to system behavior. The Conclusions section summarizes the key findings and future perspectives for incorporating microbiota into data-driven frameworks for sustainable energy transitions.

## 2. Materials and Methods

This section first describes the dataset and the preprocessing steps undertaken. Subsequently, it details the initial linear modeling approach, followed by a feature selection process based on variable weighting to derive a simplified, yet robust, model. Finally, it presents the development of an adaptive predictive model using a moving window technique combined with a regularization method to prevent overfitting.

### 2.1. Substrate Selection

The substrates used in this study were fresh PM and CD. The inoculum, obtained from the same source as the manure, was included to ensure microbial compatibility with the feedstock. Both were collected at a small-scale pig farm located in the municipality of Florida, Valle del Cauca, where approximately 20 pigs are kept under semi-intensive conditions. Animal pens are washed twice daily, and the resulting wastewater, rich in organic matter, drains into a static open-air tank that served as the inoculum source. Fresh manure was manually collected after excretion using sanitized tools. CD were obtained from a medium-sized cassava starch-processing facility located in the rural area of Mandiba, Santander de Quilichao, Cauca. Processing nearly eight tons of cassava per day, the plant generates over two tons of lignocellulosic residue each week. This material was delivered in dry, milled form.

All samples were stored at 4 °C until physicochemical characterization, which included proximate analysis by gravimetric methods and determination of the carbon-to-nitrogen (C:N) ratio via high-temperature combustion. These procedures followed the Standard Methods for the Examination of Water and Wastewater (APHA, AWWA, WEF), ensuring analytical consistency as summarized in Table 1 [31,32,33].

### 2.2. Experimental Setup

**Figure 1:**
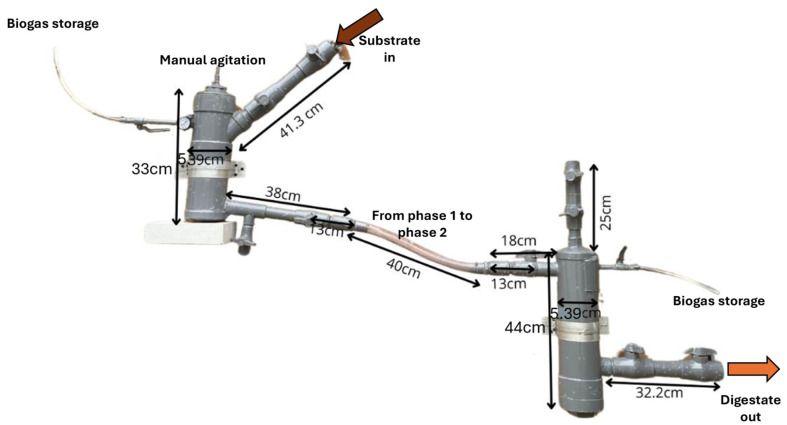
Two-phase digester made of PVC.

The experimental setup consisted of a two-phase laboratory-scale anaerobic digester designed to operate without integrated control systems Figure 1. The system was constructed using 110 mm sanitary-grade PVC tubing due to its low cost, durability, and ease of assembly. Phase 1 (D1F1) (3 L) was expected to perform hydrolysis and acidogenesis, while phase 2 (D1F2) (4 L) supposedly supported acetogenesis and methanogenesis. Each chamber was operated at 80% of its total volume, 2.4 L in phase 1 and 3.2 L in phase 2, leaving the remaining headspace for biogas accumulation. To enable real-time monitoring, a low-cost IoT module was incorporated into the digester, integrating an Arduino UNO microcontroller with sensors for pH, temperature, and methane concentration. Data was transmitted through a mobile network to the ThingSpeak platform for remote visualization [26]. This setup allowed continuous monitoring without the need for sophisticated instrumentation.

### 2.3. Operational Parameter

To establish an active MC, both phases were fed inoculum for five days, until reaching a working volume. The inoculum had a C:N ratio of 10.3 and 2.2% TS. During start-up, the OLR, estimated with a five-day HRT, was 8.37 gVS/L·day. Thereafter, feeding used a 73:27 blend of PM and CD. The daily feed was 35 g fresh PM and 13 g CD, plus 166 g water to achieve 10% TS (214 g/day total). The theoretical C:N ratio was 21.55. With the defined working volumes, HRTs were 12 days for D1F1 and 15 days for D1F2. Corresponding OLRs were 7.7 and 5.7 gVS/L·day. vs. inputs were 18.46 g/day (D1F1) and 18.45 g/day (D1F2). Daily manual feeding with graduated containers and isolation valves ensured accurate dosing and anaerobiosis.

The IoT-instrumented digester (D1) enabled incremental, data-driven feed adjustments in both phases (D1F1, D1F2) using real-time pH, temperature, and methane concentration. These signals guided when to lower the OLR and TS and when to apply temporary pH control, moving the reactors toward consistent operating conditions. Five feed formulations were implemented (Table 2). In D1F1, pH was briefly corrected with lime and then NaOH to keep it within 6.5–7.5; by mixture 5, recirculated digestate from D1F2 maintained pH without further chemicals. Mixture 4 used inoculum from an anaerobic digester at a university in Colombia treating food waste. Across mixtures, TS was reduced from 10% to 8–9%, OLR decreased from 12.4 gVS/L·day (inoculum step) to 5–6 gVS/L·day, and the C:N ratio increased in the final mixture due to recirculation while the contributions of PM and CD were reduced.

### 2.4. Steady State

Identifying steady-state periods was essential to build a reliable dataset, define representative operating conditions, and guide downstream variable prioritization and modeling. pH, temperature, and methane concentration were monitored continuously for 161 days (24/7). The IoT system logged three readings per minute for each variable and was routinely cross-checked against bench measurements to validate operational reliability.

Data volume was substantial, D1F1 recorded 694,110 samples per variable and D1F2 573,215. Processing followed six steps: (1) splitting timestamp into date and time; (2) validity filtering (e.g., pH 3–12; 10–45 °C; CH_4_ within instrument bounds) with out-of-range values set to blank; (3) multivariate imputation by chained equations (MICE) to preserve temporal continuity [34]; (4) resampling to hourly means (2893 rows in D1F1; 2389 in D1F2) and (5) to daily means (152 and 147, respectively), retaining trends while reducing computational load as shown in Table 3.

Stable windows were then identified via rolling windows using relative standard deviation thresholds (<15%) around moving means for pH, temperature, and methane concentration, with a minimum continuous duration and compliance with predefined operating limits [35]. D1 showed extended steady windows, typically with pH 6.5–7.5, facilitated by high-frequency data and the ability to adjust operating conditions in real time.

### 2.5. VFA Quantification

Samples were collected every three days in 5 mL Eppendorf tubes and stored at −20 °C until analysis. The final selection of samples for analysis was made considering the periods of system stabilization under IoT monitoring and budgetary constraints, prioritizing those most representative of the overall process behavior. Sampling was carried out during the active operation of the digester.

The quantification of VFAs was performed by gas chromatography, following the procedure described in section 5560D of the Standard Methods for the Examination of Water and Wastewater (APHA) [36], in the laboratory of the Department of Chemical Engineering and Analytical Chemistry at the University of Barcelona. Prior to chromatographic analysis, the samples were centrifuged and filtered through 0.45 µm nylon membranes to remove suspended solids. Each analysis vial contained 1 mL of sample, diluted or not depending on the estimated concentration level, along with 0.1 mL of 15% orthophosphoric acid containing a known concentration of 2-ethylbutyric acid (~500 mg/L) as an internal standard. This compound allowed verification of injection consistency and facilitated calibration of the equipment through the ratio of analyte to standard peak areas.

Analyses were carried out on a Shimadzu GC-2010 Plus (Shimadzu Corporation, Kyoto, Japan) gas chromatograph with a flame ionization detector, using a DB-FFAP capillary column, 30 m × 0.25 mm × 0.25 µm (Agilent Technologies, Santa Clara, CA, USA). The oven temperature program started at 60 °C with a two-minute hold, followed by an increase of 20 °C/min up to 240 °C, maintained for an additional two minutes. The total analysis time was 13 min. The injector (SPL-1) operated at 220 °C in split mode, with a split ratio of 50:1. Helium was used as the carrier gas at a pressure of 42.6 kPa, with a total flow of 233.4 mL/min, a column flow of 8.86 mL/min, and a linear velocity of 60 cm/s. The purge flow was set at 3 mL/min, and the makeup gas flow (nitrogen) at the detector was 10 mL/min. The injection volume was 2 mL, using helium, air, hydrogen, and nitrogen as auxiliary gases.

For equipment calibration, a commercial VFA standard (Volatile Free Acid Mix, CRM46975, Supelco/MiliporeSigma [37]) containing defined concentrations of acetic, propionic, isobutyric, butyric, isovaleric, valeric, isocaproic, caproic, hexanoic, and heptanoic acids was used. Serial dilutions were prepared in 1:1, 1:2, 1:4, 1:8, 1:16, and 1:32 ratios, to which orthophosphoric acid and the internal standard were also added. For alcohol analysis (ethanol, propanol, and butanol), defined-concentration standard solutions were prepared, applying the same dilutions and analytical conditions. This procedure allowed precise and reproducible determination of VFAs in the samples, essential for evaluating the performance of the AD system and its relationship with operating conditions and microbiota.

### 2.6. Metagenomic Analysis

Samples for metagenomic analysis were collected directly from operational biodigester using 50 mL Falcon tubes. Sampling was performed every three days throughout the process, following the same prioritization criteria used for the quantification of VFAs, focusing on periods of greatest microbiological representativeness and considering the availability of resources. Once collected, samples were immediately frozen at −20 °C and stored until further processing.

To analyze the MC, Falcon tubes were sent to Omega Bioservices (Norcross, GA, USA) for DNA extraction using the kit E.Z.N.A.^®^ Universal Pathogen Kit, library preparation and for sequencing the V3–V4 hypervariable region of the 16S rRNA gene using the primers 341F (CCTACGGGNGGCWGCAG) and 806R (GACTACHVGGGTATCTAATCC) which was conducted on an Illumina Miseq sequencing platform (Illumina, San Diego, CA, USA) (Paired-end sequencing 300 bp). Illumina reads were then analyzed using BaseSpace app (version 1.1.3) [38]. Thus, raw sequence data were demultiplexed and then quality filtered, denoised, merged, and chimera removed using the DADA2 [39] to generate amplicon sequence variants (ASVs). Taxonomic assignment was conducted using the SILVA database (version 138.2) [40].

To structure the analysis of microbial interactions, a subset of phyla of interest was defined from the general metagenomic dataset, considering the sequencing reads obtained for each taxonomic group. The selection was based on two main criteria. First, the sustained presence of each phylum throughout the monitoring period was evaluated, excluding those with very low or intermittent representation, as their variability would hinder the detection of consistent associations in the relational analysis. Second, functional relevance reported in previous studies on anaerobic digestion was reviewed, prioritizing phyla whose involvement in fermentative, acetogenic, or methanogenic pathways has been extensively documented in similar systems [7,41].

Once the representative periods were defined, the results from VFA quantification and metagenomic analysis were integrated, extending the characterization to the biochemical and microbiological components of the system. In several cases, the observed patterns were consistent with those reported in the specialized literature, which supported the robustness of the approach. The dataset included operational, biochemical, and microbiological variables [42,43,44,45,46].

Since the biochemical and microbiological measurements were less frequent than the operational records, imputation techniques were applied within the selected periods to expand the dataset without distorting the relationships among variables. Methods such as KNN imputation, iterative imputation, and MICE were employed [43,47]. The analysis focused on the period between days 97 and 154, which, although not representing a fully stabilized phase, shows a trend toward stabilization and coincides with the selected VFA and microbiological samples. This ensured consistency between the experimental data and the operational conditions.

### 2.7. Preprocessing and Unified Database

Once the representative periods were defined, the results from VFA quantification and metagenomic analysis were incorporated to extend the characterization of the system to its biochemical and microbiological dimensions. The patterns obtained aligned with those reported in specialized literature, reinforcing the validity of the approach [42,43,44,45,46]. The unified dataset combined operational, biochemical, and microbiological variables. Because biochemical and microbiological measurements were less frequent than operational records, imputation method MICE was applied to harmonize the dataset without altering the underlying relationships among variables [43,47].

**Figure 2:**
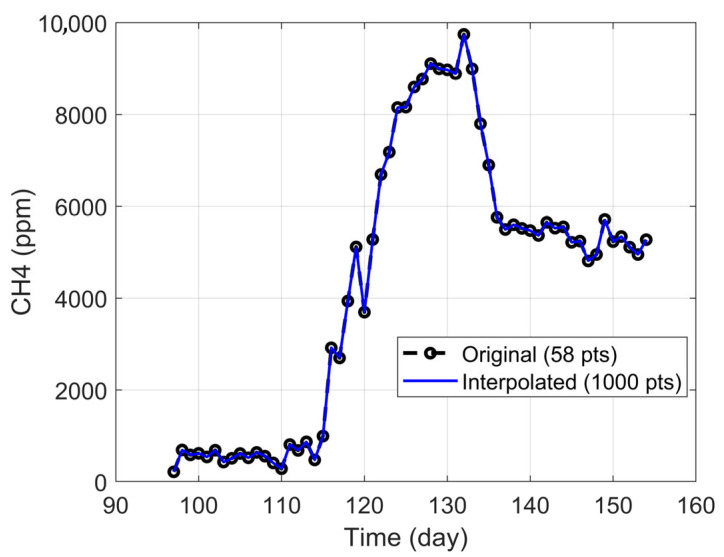
Original (58 points) vs. interpolated (1000 points) time-series data for CH_4_ concentration.

The analysis focused on the period between days 97 and 154, which, while not fully stabilized, displayed a clear trend toward steady performance and coincided with the VFA and microbiological samples selected. This ensured coherence between experimental observations and operational conditions. The resulting dataset comprised daily averages over 58 days, which were further refined through linear interpolation to increase temporal resolution. This process expanded the series to 1000 points, enabling the application of moving window analyses, as illustrated in Figure 2.

The interpolation was validated for all variables, yielding R^2^ values close to 1 and the mean relative error (MRE) values around 0.1%, confirming a high-fidelity representation of the original data.

### 2.8. Linear Modeling

To simplify the proposed equations and procedure, the suffixes associated with each VFA (Table 4) microorganism (Table 5), and operating condition (Table 6) are shown below.

Equation (1) that linearly approximates $CH_{4}$ concentration as a function of the microorganisms, fatty acids, and operating conditions was proposed in the following linear form, based on the suffixes from Table 4, Table 5 and Table 6.

(1) $${\left(CH_{4}\right)}_{aprox}=C_{1}\left(a_{1}\right)+C_{2}\left(a_{2}\right)+\ldots +C_{25}\left(m_{25}\right)+C_{26}\left(m_{26}\right)+\ldots +C_{30}\left(p_{30}\right)+C_{31}\left(p_{31}\right)$$

In its matrix form (matrix $\underline{A}$), Equation (1) can be expressed as follows (Equation (2)):

(2) $$\left[\begin{array}{c} {\left(CH_{4}\right)}_{1}\\ {\left(CH_{4}\right)}_{2}\\ \begin{array}{c} .\\ .\\ .\\ {\left(CH_{4}\right)}_{n} \end{array} \end{array}\right]=\left[\begin{array}{ccc} {\left(a_{1}\right)}_{1} & {\left(a_{2}\right)}_{1} & \begin{array}{cc} \ldots & \begin{array}{ccc} {\left(m_{26}\right)}_{1} & \ldots & {\left(p_{31}\right)}_{1} \end{array} \end{array}\\ {\left(a_{1}\right)}_{2} & {\left(a_{2}\right)}_{2} & \begin{array}{cc} \ldots & \begin{array}{ccc} {\left(m_{26}\right)}_{2} & \ldots & {\left(p_{31}\right)}_{2} \end{array} \end{array}\\ \begin{array}{c} .\\ .\\ .\\ {\left(a_{1}\right)}_{n} \end{array} & \begin{array}{c} .\\ .\\ .\\ {\left(a\right)}_{n} \end{array} & \begin{array}{c} .\\ .\\ .\\ \begin{array}{cc} \ldots & \begin{array}{ccc} {\left(m_{26}\right)}_{n} & \ldots & {\left(p_{31}\right)}_{n} \end{array} \end{array} \end{array} \end{array}\right]\left[\begin{array}{c} C_{1}\\ C_{2}\\ \begin{array}{c} .\\ .\\ .\\ C_{31} \end{array} \end{array}\right]$$

where the constants $C_{i}$ are the approximation coefficients. This matrix form from Equation (2) can be written more compactly as shown in Equation (3):

(3) $$\underline{CH_{4}}=\underline{A}\;\underline{x}$$

The matrix $\underline{A}$ contains data collected from fatty acids, microorganisms, and operating conditions, the vector $\underline{CH_{4}}$ represents the collected methane production data, while the vector $\underline{x}$ contains the approximation coefficients that must be determined to formulate the model. The vector $\underline{x}$, can be solved by rearranging Equation (3) as follows:

(4) $$\underline{x}=[{\underline{A}}^{T}\underline{A}{]}^{-1}{\underline{A}}^{T}\underline{CH_{4}}$$

where ${\underline{A}}^{T}$ is the transpose of matrix $\underline{A}$.

#### 2.8.1. Assessing Variable Importance

To determine the relative importance of each variable in the approximation, and subsequently define a smaller, more practical subset (as working with all 31 variables can be impractical and costly in terms of laboratory testing), a variable weighting method was used. Therefore, Equation (5) appears as a modification of Equation (3) considering the minimal error ϵ.

(5) $$\underline{CH_{4}}=\underline{A}\;\underline{x}+\epsilon$$

To quantify how much each variable “contributes” to the $CH_{4}$ production within the approximation, it is necessary to measure the relevance of each variable in the linear model. Since each variable may be measured on a different scale (e.g., microorganism abundance vs. fatty acid concentration in mg/L), directly comparing the raw coefficients $C_{i}$ in vector $\underline{x}$ can be misleading. Therefore, it is necessary to standardize the input data. In the same way, to compare the relative importance of each variable, the coefficients $C_{i}$ that form the vector $\underline{x}$ were standardized (as z-scores). The standardized coefficient $C_{i}^{*}$ for each variable $C_{i}$ was calculated as:

(6) $$C_{i}^{*}=C_{i}\frac{\sigma_{C_{i}}}{\sigma_{CH4}}$$

where $\sigma_{C_{i}}$ and $\sigma_{CH4}$ are the standard deviations of the approximation coefficient $C_{i}$ and the response variable $CH_{4}$, respectively.

#### 2.8.2. Predictive Model

To capture the evolutionary nature of the anaerobic digestion process, a dynamic predictive model was developed based on a moving windows approach. The model operates iteratively. At each time step t, a linear regression model is trained using a window containing the last k observations (in this case, k=10 was chosen). This model is then used to make a one-step-ahead prediction of $\underline{CH_{4}}$ (denoted as $\underline{\hat{CH_{4}}}$), as a function of the weighted variables previously described. However, the use of small data windows can lead to overfitting. To address this problem and improve the model’s generalization capability, Ridge Regression was used instead of ordinary least squares. This regression introduces a penalty term into the least squares cost function. For each time window, the objective is to find the coefficient vector $\underline{x}$ that minimizes the following function:

(7) $${min}_{\underline{x}}\left({\left\|\underline{CH_{4}}-\underline{A}\;\underline{x}\right\|}_{2}^{2}+\lambda {\left\|\underline{x}\right\|}_{2}^{2}\right)$$

where ${\left\|\underline{CH_{4}}-\underline{A}\;\underline{x}\right\|}_{2}^{2}$ is the sum of squared errors (the data fit term at time t), $\lambda {\left\|\underline{x}\right\|}_{2}^{2}$ is the regularization term applied at time t, λ is the regularization hyperparameter that controls the balance between the data fit and the model simplicity, and $\underline{x}$ contains the coefficients $C_{i}$. The hyperparameter λ was selected to improve the model predictive performance (λ=2). Thus, Equation (4) is rewritten to obtain the predictive parameters ($\underline{\hat{x}}$) by solving the following equation:

(8) $${\left(\underline{\hat{x}}\right)}_{t+1}={\left([{\underline{A}}^{T}\underline{A}+\lambda \underline{I}{]}^{-1}{\underline{A}}^{T}\underline{CH_{4}}\right)}_{t}$$

where $\underline{I}$ is the identity matrix. The goal of Equation (8) is to find the value of the coefficients in vector $\underline{x}$ at time t+1 using the data available at time t. Using Equation (8), it is possible to find the $\underline{\hat{CH_{4}}}$ values for a subsequent window, given a defined window size of k=10. In this way, Equation (3) becomes a prediction equation as follows:

(9) $${\left(\hat{\underline{CH_{4}}}\right)}_{t+1}={\underline{\left(A\right)}}_{t+1}({\hat{\underline{x}})}_{t+1}$$

#### 2.8.3. Model Performance Evaluation

The precision of the predictive model was quantified using three standard statistical metrics. These metrics evaluate the divergence between the real observed values of $\underline{CH_{4}}$ and the values predicted by the model, $\hat{\underline{CH_{4}}}$. On one hand, the Coefficient of Determination ($R^{2}$) indicates the proportion of the variance in methane production that is predictable from the independent variables. A value close to 1 indicates an almost perfect fit. Equation (10) shows how it was calculated for this case.

(10) $$R^{2}=1-\frac{\sum_{i=1}^{n}{\left[{\left(CH_{4}\right)}_{i}-{\left(\hat{CH_{4}}\right)}_{i}\right]}^{2}}{\sum_{i=1}^{n}{\left[\left({\left(CH_{4}\right)}_{i}-\bar{CH_{4}}\right)\right]}^{2}}$$

Next, the Root Mean Square Error (RMSE) represents the standard deviation of the prediction residuals. It is a measure of the average error of the model in the same units as the response variable (ppm of $CH_{4}$), which facilitates its interpretation and is expressed in Equation (11).

(11) $$RMSE=\sqrt{\frac{1}{n}\sum_{i=1}^{n}{\left[{\left(CH_{4}\right)}_{i}-{\left(\hat{CH_{4}}\right)}_{i}\right]}^{2}}$$

Finally, the MRE measures the average error in relative or percentage terms with respect to the real value is defined in Equation (12). The absolute value was used to prevent positive and negative errors from canceling each other out.

(12) $$MRE=\frac{1}{n}\sum_{i=1}^{n}\left|\frac{{\left(CH_{4}\right)}_{i}-{\left(\hat{CH_{4}}\right)}_{i}}{{\left(CH_{4}\right)}_{i}}\right|$$

In Equations (10)–(12), ${\left(CH_{4}\right)}_{i}$ is the real value of the i-th observation, ${\left(\hat{CH_{4}}\right)}_{i}$ is the value predicted by the model for the i-th observation, $\bar{CH_{4}}$ is the mean value of all real values, and n is the total number of observations used for the evaluation.

## 3. Results and Discussion

### 3.1. Digester Performance

During the implementation of the two-phase biodigester, one of the main technical challenges was controlling gas leaks and internal pressure, which required multiple structural adjustments and caused delays in the early stages of operation. The installed manometers failed, likely due to H_2_S-induced corrosion, while the valves progressively stiffened with use, in some cases requiring replacement. In addition, certain PVC weld joints developed fractures, compromising system integrity. The manual stirring mechanism did not improve process performance but was associated with gas leaks, leading to its deactivation and reinforcement of seals. After several corrective interventions, continuous and functional operation was achieved for the duration of the experiment.

#### 3.1.1. IoT Monitoring Advantages

Although low-cost systems might be unsuited for long-term use or high-precision data collection, these biodigesters provide a practical alternative for experimental applications at laboratory scale when resources are limited. The estimated cost of assembling a two-phase biodigester without IoT monitoring was 300 USD, while the addition of a digital monitoring system increased the total to 420 USD per unit. Although some sensors required replacement during operation, the IoT system performed reliably, providing consistent readings comparable to manual instruments. Its implementation enabled real-time, continuous data acquisition, which was essential for detecting operational variations, making timely adjustments, and improving process understanding.

**Figure 3:**
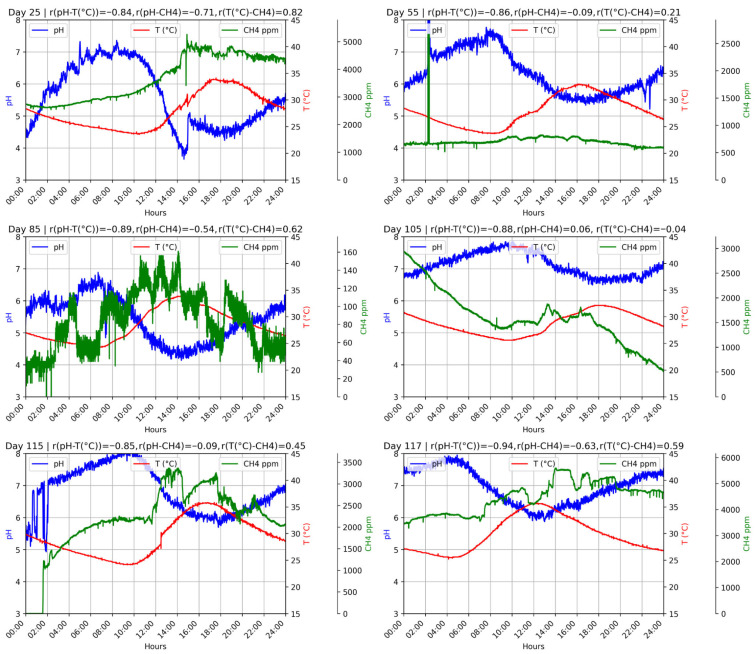
D1F1 operation during 24 h on random days.

**Figure 4:**
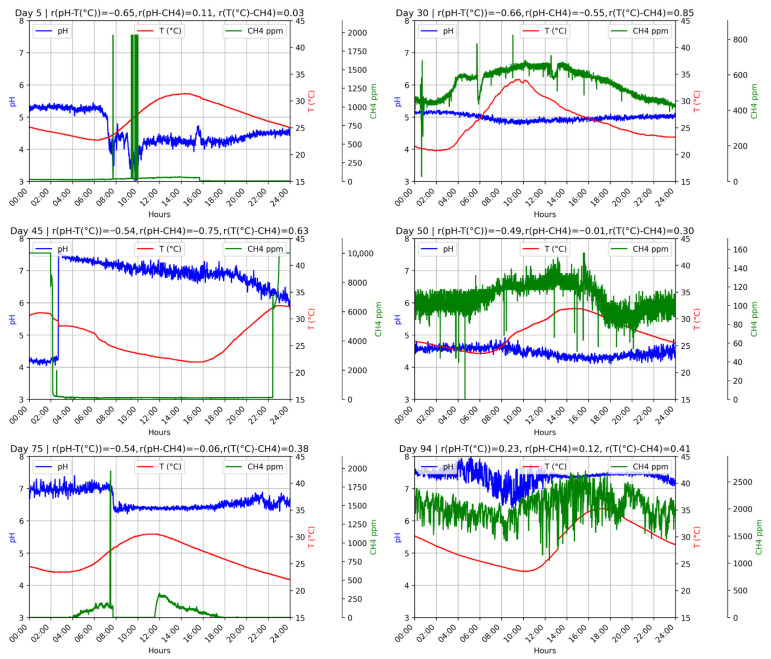
D1F2 operation during 24 h on random days.

Figure 3 (D1F1) and Figure 4 (D1F2) show the 24 h profiles of pH, temperature, and CH_4_ concentration during different operational stages. The days were randomly selected from both phases to provide representative snapshots of system behavior under varying conditions. In all cases a consistent inverse relationship was observed, where pH increased during early morning hours as ambient temperature declined and then decreased progressively as temperature rose throughout the day. In D1F1 this trend was stronger and more reproducible, with correlation coefficients between −0.84 and −0.94, while in D1F2 the association was weaker (between −0.49 and −0.66) and accompanied by larger fluctuations in methane concentrations. These results highlight the direct effect of ambient thermal oscillations on microbial activity, especially on pH dynamics.

This behavior may be linked to phases of microbial adaptation or to the accumulation of internal self-regulation mechanisms. As temperature dropped during the night and metabolic activity slowed, nitrogenous compounds likely continued decomposing and releasing ammonia (NH_3_). This ammonia could react with dissolved CO_2_, which is more soluble at low temperatures, to form ammonium bicarbonate (NH_4_HCO_3_). The resulting increase in alkalinity buffered pH variations, preventing excessive acidification and contributing to system resilience [48,49].

Such dynamics are rarely captured in conventional laboratory-scale digesters where data are typically restricted to discrete measurements. In this case the use of IoT-based continuous monitoring provided hourly resolution and made it possible to identify fine-scale responses such as the rise in pH at lower nighttime temperatures that would otherwise remain unnoticed. This approach delivers a more realistic picture of system performance under environmental conditions and emphasizes the value of continuous monitoring strategies for interpreting anaerobic digestion behavior beyond the limits of punctual sampling.

#### 3.1.2. Stabilization of Anaerobic Codigestion

**Figure 5:**
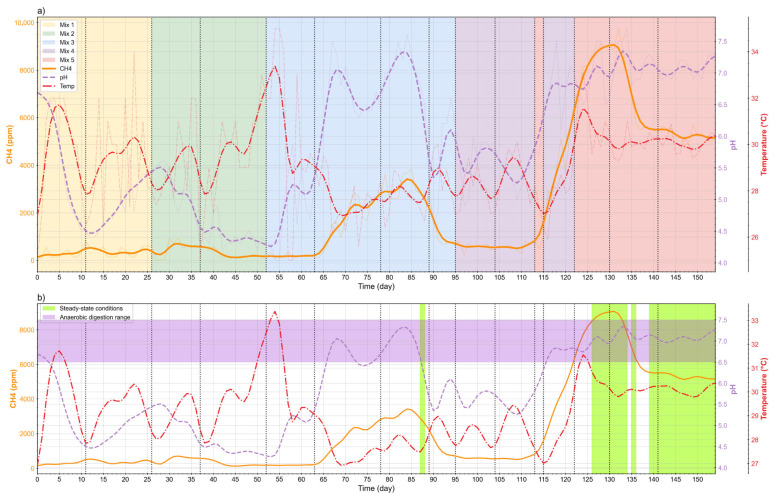
(**a**) Mixture feeding over time (**b**) Steady-state identification.

Achieving a steady-state is a critical milestone in AD, as it reflects the convergence of operational and microbial conditions that support sustained methanogenic activity [50,51]. In system D1, specific time segments were identified where pH, temperature, and methane concentration aligned within the functional ranges expected for AD. Figure 5 integrates these three variables across the full operational period, providing a comprehensive view of the transitions from unstable to stabilized phases. This visualization not only highlights the progression of the system under different operating conditions but also illustrates how corrective measures and phase-specific dynamics gradually steered the reactor toward a functional equilibrium.

In Figure 5a, a shift becomes evident after day 120, when pH consistently remained above 6.5 while methane rose steadily, surpassing 8000 ppm by day 132. These conditions coincided with stable temperatures between 30 and 32 °C, an optimal mesophilic range that favors methanogenic activity [35,42]. The segmentation into D1F1 and D1F2 reveals the influence of each phase under a combined HRT of 27 days. During early mixtures (Mix1–Mix2), high OLR and the absence of adapted inoculum produced irregular methane signals dominated by acidogenesis. From Mix3 onwards, corrective measures such as alkalinization promoted higher CH_4_ concentrations, although fluctuations beyond ±15% prevented these periods from being classified as steady. Toward Mix4 and Mix5, adjustments including higher inoculum input and recirculation from D1F2 likely increased microbial density and functional diversity, progressively creating conditions more favorable to methanogenesis [52,53].

Figure 5b highlights two segments where the system approached steady-state behavior. The first, between days 126 and 136, was characterized by pH values between 6.5 and 7.5, methane above 8000 ppm, and stable temperatures (30–32 °C), all within functional ranges and with fluctuations below ±15%. A temporary decline in methane around day 137 disrupted this stage, but from day 139 onwards the system recovered, initiating a second steady segment that persisted until the end of the experiment.

Overall, the convergence of pH, CH_4_, and temperature demonstrates that the steady state achieved in D1 was not the result of a single correction but the cumulative effect of progressive adjustments. This sequence of changes allowed the system to transition from acidogenic predominance to a consolidated methanogenic phase, representing a functional stabilization consistent with the goals of two-phase AD [53].

### 3.2. Volatile Fatty Acids (VFAs) and Metagenomic Analysis

Once stabilization was established from IoT-monitored variables, VFAs and microbiota were analyzed during the transition toward optimal operation (days 97–154, Mix 4 and Mix 5). Thirteen samples were taken, and missing data were inputted through MICE to ensure continuity.

**Figure 6:**
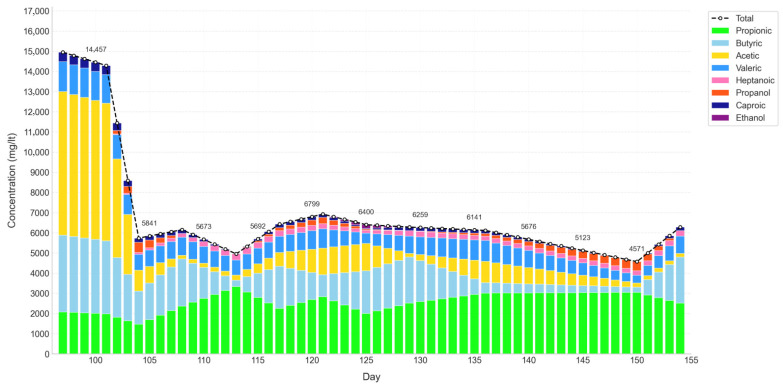
VFA and alcohol concentration in D1.

Figure 6 displays when VFA concentrations dropped sharply after day 100, from above 14,000 mg/L to 5800 mg/L, before oscillating between 5000 and 7000 mg/L. This decline reflects the mitigation of acidogenic pressure and the progressive adjustment of the microbial community, setting conditions increasingly suitable for methanogenesis and linking metabolite dynamics with microbial responses in the path toward functional balance [54,55]. 

The individual analysis of VFAs confirmed that the steep decline after day 100 was largely driven by the reduction in acetic and butyric acids, both tied to early fermentative pathways [54]. Between days 103 and 118, however, propionic acid and medium-chain carboxylates (C5–C8), including caproic, heptanoic, and valeric, increased notably, reaching averages of 140 mg/L, 218 mg/L, and 820 mg/L, respectively [56]. These less common metabolites are typically linked to secondary fermentation processes or to transitional phases of temporary accumulation [57,58,59]. Their persistence, together with measurable levels of propanol (134 mg/L) and the absence of ethanol, suggests a fermentative stage dominated by chain-elongation routes, potentially hindered by propanol’s inhibitory effect on methanogenic consortia [57,60,61]. After day 119, these acids gradually declined (e.g., caproic down to 109 mg/L, valeric to 758 mg/L), while ethanol reappeared (20 mg/L) and propanol rose to 191 mg/L. This pattern may indicate that, despite higher alcohol concentrations, microbes capable of degrading medium-chain acids regained activity, backing a functional shift toward methanogenesis [62,63].

**Figure 7:**
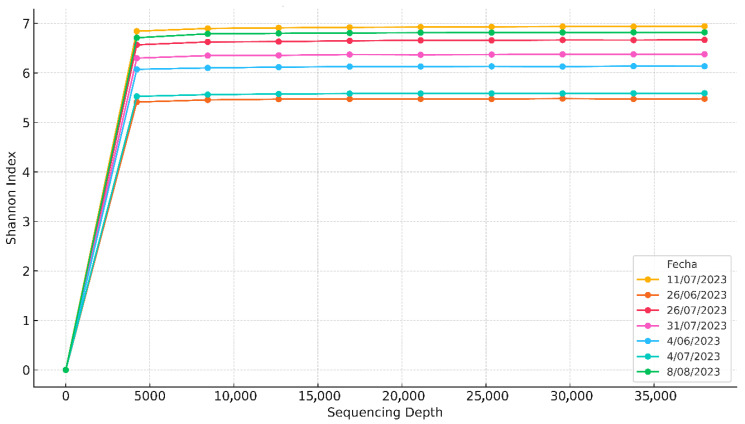
Alpha rarefaction curves of the alpha diversity (Shannon) of the 16S rRNA gene.

Regarding microbial analysis, a total of 1,815,465 high-quality reads, with an average of 201,718 ± 103,945 reads per sample. Rarefaction analysis based on Shannon index showed that sequencing depth was adequate to capture most of the bacterial diversity across samples as shown in Figure 7.

**Figure 8:**
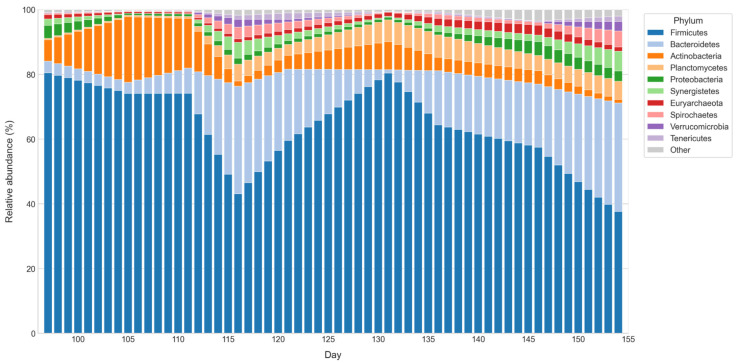
Temporal dynamics of microbial phyla in D1.

The microbial dynamics derived from phylum-level aggregate counts (Supplementary Table S1), as depicted in Figure 8, may offer insights into the associations between dominant phyla, methane concentrations, and the VFAs profiles presented in Figure 5 and Figure 6, respectively. During days 97 and 154, *Firmicutes* remained the prevailing group, averaging 49,235 reads (64.2% of the total), underscoring its central role in the early stages of the process, particularly in hydrolysis and acidogenesis [7]. This activity likely promoted the production of fermentative precursors, consistent with the elevated concentrations of acetic, propionic, and butyric acids recorded at the beginning of this interval [54]. Along with *Firmicutes*, *Bacteroidetes* (15%) and *Actinobacteria* (7.5%) contributed to medium-chain fatty acids such as valeric and caproic during the accumulation phase (days 103–118) [57,61]. This functional diversity points to a bacterial consortium engaged in degrading complex polymers and extending fermentative pathways, buffering intermediates before methanogenic activity resumed [64,65]. Toward the end, *Firmicutes* declined while *Euryarchaeota* increased to 1.5%, coinciding with reduced VFAs and steadier methane, suggesting activation of acetoclastic and hydrogenotrophic routes [7,66].

Minor groups might have played complementary roles, with *Planctomycetes* (3.9%), likely coupling sulfide oxidation to methanogenesis, *Proteobacteria* (1.9%) contributing to propionate and acetate turnover, and *Synergistetes* (1.9%) participating in syntrophic H_2_ transfer [7,67]. Unexpectedly, *Verrucomicrobia* appeared in the community profile, a phylum typically restricted to volcanic habitats dominated by acidophilic methanotrophs [68]. These bacteria can oxidize methane as their main substrate and, to a lesser extent, hydrogen, carbon dioxide, ammonium and hydrogen sulfide, functioning as natural biofilters in extreme ecosystems. Their presence in a mesophilic anaerobic digester is unusual and may reflect residual inoculum or localized microredox niches rather than an active role in methanogenesis [68,69]. Alongside other low-abundance phyla such as *Lentisphaerae*, *Candidatus saccharibacteria*, and *Parcubacteria*, their detection expands the taxonomic spectrum and raises questions about potential ecological roles still unexplored in anaerobic bioenergy systems [70]. Many of these groups remain unresolved at the species level, even after advanced genomic assembly, forming part of the so called microbial dark matter. This hidden fraction highlights one of the major challenges in deciphering the functional complexity of anaerobic microbiomes [71,72].

### 3.3. Multiple Linear Regression (MLR)

Modeling phase was based on the Supplementary Table S2. The values in Table 7 show the coefficients $C_{i}$ obtained by finding the vector $\underline{x}$ after applying Equation (4).

**Figure 9:**
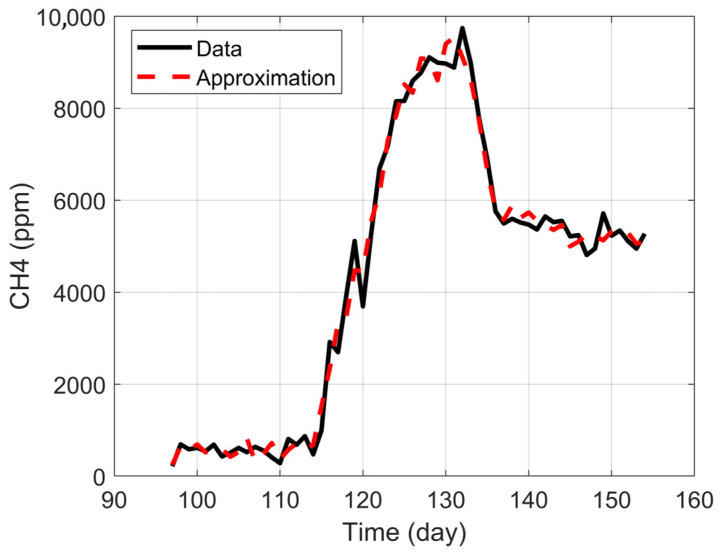
Approximation result.

This method of finding the approximation coefficients is known by some authors as inverse modeling and can be considered a multilinear regression. In fact, this inverse modeling approach is equivalent to the least squares method applied to multiple vectors. When the vector $\underline{x}$ was obtained, Equation (1) was applied to generate the approximation curve. Figure 9 shows the resulting approximation.

#### 3.3.1. Data Prioritization

**Figure 10:**
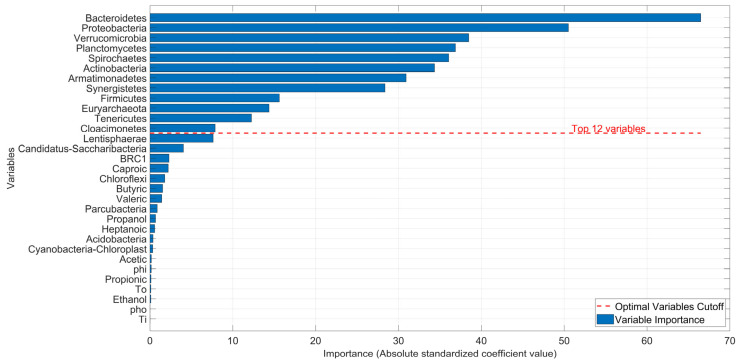
Relative variable importance with defined cut-off.

While Figure 9 shows the overall approximation result, it is important to determine the contribution of each fatty acid, microorganism, or operating condition to the $CH_{4}$ production. As previously mentioned, a direct comparison of the $C_{i}$ coefficients can be misleading, so it is important to perform a variable weighting process using Equation (6). The values obtained from this process are shown in Table 8 and plotted in Figure 10.

**Figure 11:**
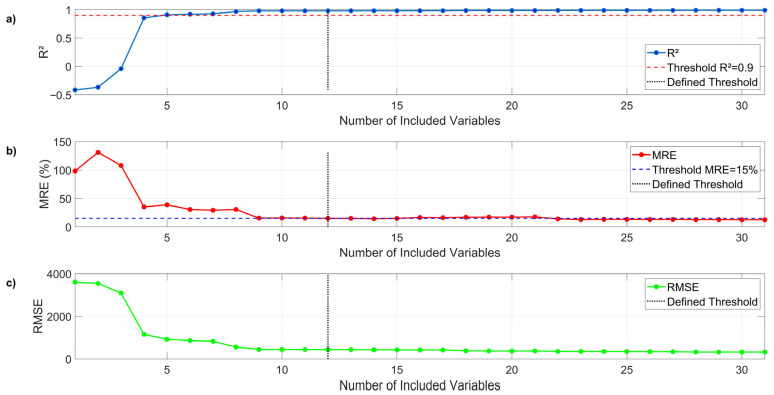
(**a**) R^2^, (**b**) MRE and (**c**) RMSE vs. Number of included variables in the approximation.

To define how many variables are needed to achieve good fit without a significant loss of precision, two limits were established, as can be seen in Figure 11, with an $R^{2}$ Coefficient greater than 0.9 and an MRE lower than 15%. Based on Table 8 and Figure 10, the 12 variables with the highest values, or greatest impact on $CH_{4}$ production, were selected. They were $C_{10}^{*}$, $C_{12}^{*}$, $C_{17}^{*}$, $C_{13}^{*}$, $C_{15}^{*}$, $C_{11}^{*}$, $C_{20}^{*}$, $C_{14}^{*}$, $C_{9}^{*}$, $C_{16}^{*}$, $C_{19}^{*}$ and $C_{18}^{*}$. According to Table 5, these correspond to *Bacteroidetes*, *Proteobacteria*, *Verrucomicrobia*, *Planctomycetes*, *Spirochaetes*, *Actinobacteria*, *Armatimonadetes*, *Synergistetes*, *Firmicutes*, *Euryarchaeota*, *Tenericutes*, and *Cloacimonetes*, correspondingly.

The predictive weight of the identified phyla by the model is substantiated by their established functional roles in AD. Key bacterial groups such as *Bacteroidetes, Proteobacteria, Actinobacteria*, and *Spirochaetes* are recognized for their indispensable roles in the hydrolysis and acidogenesis stages. These phyla collaborate to break down complex organic matter into direct precursors for methanogenesis, including acetate and $H_{2}$ [44,73]. As expected, the phylum *Euryarchaeota*, which encompasses all known methanogens, was a fundamental predictor, being responsible for the final conversion to methane [74,75]. Notably, the model also highlighted the importance of *Verrucomicrobia*, a finding validated by research demonstrating that members of this phylum can actively degrade complex polysaccharides like xylan under anaerobic conditions, positioning them as specialized primary degraders [76]. Altogether, these results confirm that the model successfully captures the complex microbial network that drives methane production, from the initial decomposers to the terminal methanogens.

Thus, Equations (1) and (2) can be rewritten in terms of the 12 variables that were found to be most important according to the weighting performed. This leads to Equation (13):

(13) $$\begin{array}{cc} {\left(CH_{4}\right)}_{WeightedAprox} & \\ & =C_{10}\left(m_{10}\right)+C_{12}\left(m_{12}\right)+C_{17}\left(m_{17}\right)+C_{13}\left(m_{13}\right)+C_{15}\left(m_{15}\right)\\ & +C_{11}\left(m_{11}\right)+C_{20}\left(m_{20}\right)+C_{14}\left(m_{14}\right)+C_{9}\left(m_{9}\right)+C_{16}\left(m_{16}\right)\\ & +C_{19}\left(m_{19}\right)+C_{18}\left(m_{18}\right) \end{array}$$

In its matrix form, Equation (13) can be expressed as follows:

(14) $$\left[\begin{array}{c} {\left(CH_{4}\right)}_{1}\\ {\left(CH_{4}\right)}_{2}\\ \begin{array}{c} .\\ .\\ .\\ {\left(CH_{4}\right)}_{n} \end{array} \end{array}\right]=\left[\begin{array}{ccc} {\left(m_{10}\right)}_{1} & {\left(m_{12}\right)}_{1} & \begin{array}{cc} \ldots & {\left(m_{18}\right)}_{1} \end{array}\\ {\left(m_{10}\right)}_{2} & {\left(m_{12}\right)}_{2} & \begin{array}{cc} \ldots & {\left(m_{18}\right)}_{2} \end{array}\\ \begin{array}{c} .\\ .\\ .\\ {\left(m_{10}\right)}_{n} \end{array} & \begin{array}{c} .\\ .\\ .\\ {\left(m_{12}\right)}_{n} \end{array} & \begin{array}{c} .\\ .\\ .\\ \begin{array}{cc} \ldots & {\left(m_{18}\right)}_{n} \end{array} \end{array} \end{array}\right]\left[\begin{array}{c} C_{10}\\ C_{12}\\ \begin{array}{c} .\\ .\\ .\\ C_{18} \end{array} \end{array}\right]$$

**Figure 12:**
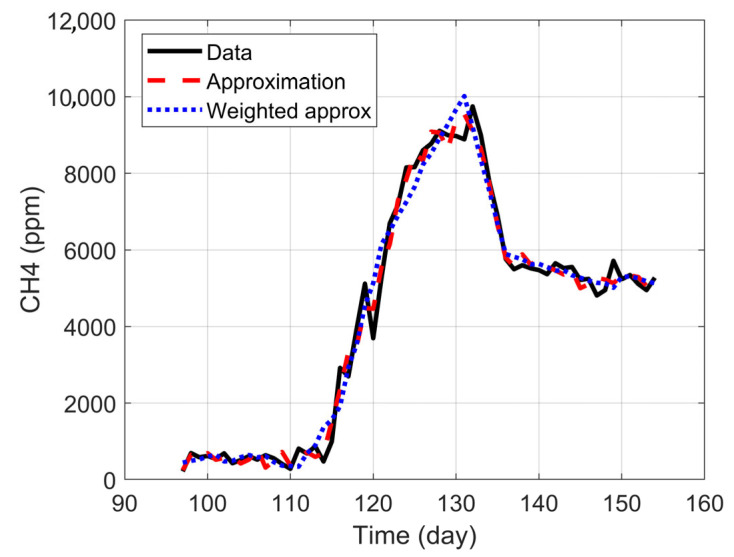
Weighted approximation.

Table 9 shows the new values found for the approximation coefficients, calculated using Equation (13) in conjunction with Equation (4). These results in the weighted approximation shown in Figure 12.

Meanwhile, the MRE for the approximation with Equation (1) is around 12.59%, while for the weighted approximation with Equation (13), it is 14.94%. Finally, the errors evaluated by the RMSE are below 450 ppm, which, considering the scale of Figure 5, are within an acceptable range. This approach successfully developed a simplified, dynamic model for predicting methane ($CH_{4}$) production in an anaerobic digestion process. The key achievement was the ability to reduce a complex system of 31 variables to a robust predictive model based on only the 12 most influential factors, without a significant loss of precision.

Table 10 shows a comparison of the metrics used to compare the fits discussed previously. While the general behavior is replicated by both curves, the $R^{2}$ coefficient for the approximation curve using Equation (1) is 0.989, whereas with the weighted approximation from Equation (13), the $R^{2}$ value is 0.979.

The primary finding of this work is the overwhelming importance of microbial populations as indicators of $CH_{4}$ concentration compared to VFAs and operational parameters. The variable weighting analysis revealed that the 12 most significant variables were exclusively microorganisms, with groups like *Bacteroidetes* and *Proteobacteria* showing the highest importance scores. This suggests that, within the context of this study, the state of the microbial community is a more direct and powerful predictor of methanogenic activity than the concentration of intermediate substrates (VFAs) or the operational conditions measured. While VFAs are essential for methanogenesis, their concentrations can be transient. In contrast, the abundance of specific microbial groups likely represents the metabolic potential of the system, making them more robust indicators for modeling purposes.

#### 3.3.2. Predictive Model Development

The simplification of the model from 31 variables to 12 demonstrates the practical value of the feature selection process. The weighted approximation model, using only the selected microorganisms, achieved an $R^{2}$ of 0.979, a negligible decrease from the 0.989 $R^{2}$ of the full model. By focusing only on the most critical microbial indicators, laboratory testing and data analysis efforts can be substantially reduced while still maintaining a high degree of accuracy. The slight increase in MRE and RMSE is an acceptable trade-off for the considerable reduction in model complexity.

**Figure 13:**
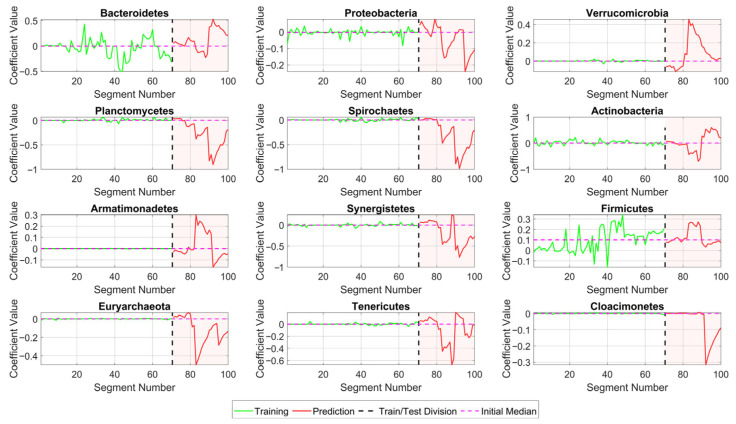
Behavior of the coefficients associated with the weighted variables.

In this context, using Equation (13), it was possible to develop a predictive model to predict the components of the vector $\underline{\hat{x}}$ and, subsequently, the behavior of the $\underline{\hat{CH_{4}}}$ production by applying Equations (8) and (9), respectively. This was performed by using 70% of the dataset to train the model and the remaining 30% to test its performance. Thus, Figure 13 shows how, by applying Equation (8), it is possible to obtain a prediction for the behavior of each of the coefficients for the weighted variables $\underline{\hat{x}}$ (originally listed in Table 9).

**Figure 14:**
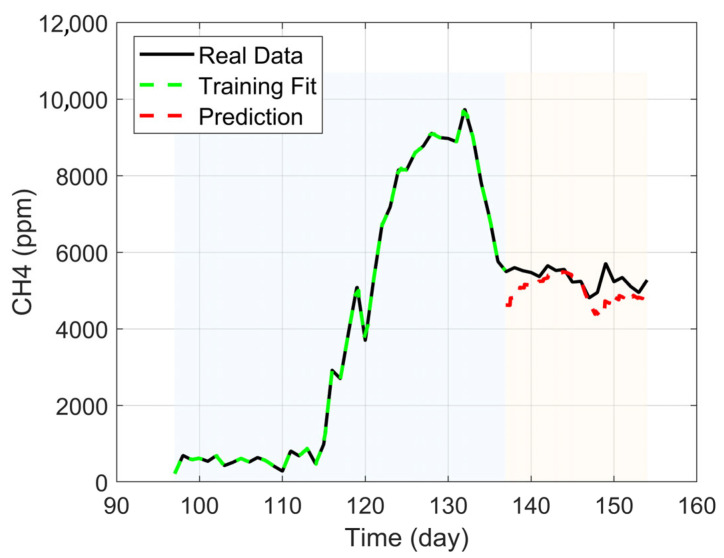
Methane prediction and training data.

By applying Equation (9), the prediction for $\hat{CH_{4}}$ was obtained, as shown in Figure 14. This was derived from the coefficients $\underline{\hat{x}}$ obtained via Equation (8). The analysis of the data from the training and prediction curves is recorded in Table 11. The $R^{2}$ fit values show a strong correlation (greater than 0.9), and the MRE values are considerably low (less than 7%), with a comparatively low RMSE for the prediction.

The development of a dynamic predictive model using a moving window approach combined with Ridge Regression proved to be highly effective. This strategy was designed to capture the evolutionary nature of the biological process and to prevent overfitting that can occur with small data windows [77]. The performance of the final predictive model on the test data was significant, achieving a coefficient of determination ($R^{2}$) of 0.920 and a MRE of 6.50%. This result confirms that the model not only fits the training data well but also generalizes effectively to make accurate short-term predictions on unseen data. The use of Ridge Regression (λ=2) was crucial in stabilizing the coefficients and ensuring the model robustness.

Several studies have also attempted to predict CH_4_ concentration in AD systems using different modeling techniques (Table 12). These works vary notably in terms of model complexity, data requirements, and analytical focus.

For instance, some approaches achieve high accuracy with computationally intensive “black-box” models, such as a Multilayer Perceptron (MLP) neural network optimized with metaheuristics like the Evaporation-Rate Water Cycle Algorithm (ERWCA) [50]. In contrast, other studies leverage massive, high-frequency Supervisory Control and Data Acquisition (SCADA) data [78], concluding that microbial inputs are unnecessary for their predictions. While research using algorithms like Random Forest (RF) confirms that combining genomic and operational data improves accuracy [44], the present study demonstrates that the microbial community structure alone can be the primary predictive driver. The MLR model developed here distinguishes itself by prioritizing a reduced set of key microbial predictors within a simpler, more interpretable framework.

This comparison underscores the value of an approach that provides a practical and computationally efficient solution, particularly for systems where microbial dynamics, rather than extensive operational data, are the main drivers of performance. The strength of this proposed model lies in its focus on microbial data. While other approaches successfully use operational data alone such as high-frequency SCADA feeds or MSW loads [30], this study demonstrates that a biological signature can be effectively employed to predict methane concentration patterns in AD. This highlights the crucial role of metagenomics in uncovering the core drivers of anaerobic digestion, enabling accurate predictions by focusing directly on the process biology.

**Figure 15:**
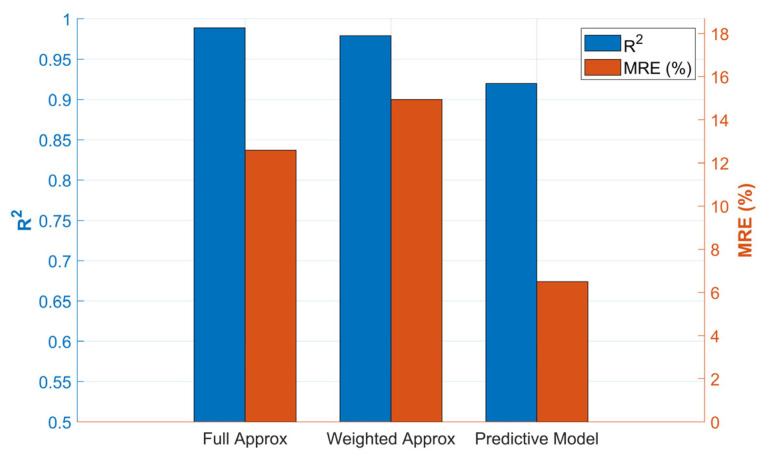
Performance comparison of the developed models.

To provide a clear summary of the study outcomes, Figure 15 visually compares the performance of the developed model variations.

The initial fitting models, the Full Approx using all 31 variables and the Weighted Approx using the 12 most important phyla, both achieved an outstanding fit on the training data ($R^{2}$ > 0.97). This confirms that the variable reduction step successfully produced a simpler model without a significant loss of explanatory power.

More importantly, the predictive model, when evaluated on the unseen test dataset, demonstrated exceptional generalization. Although its $R^{2}$ of 0.92 is slightly lower than that of the fitting models, it achieved a MRE of only 6.50%, which is less than half the error of the full approximation. This combination of a high $R^{2}$ and a significantly lower MRE on test data supports the robustness of the model and confirms the effectiveness of the proposed approach.

The novelty of this work lies in integrating MC data into an MLR framework, a significant departure from conventional modeling strategies. While previous MLR models have successfully predicted biogas production, they have predominantly relied on operational and physicochemical variables. Furthermore, studies that do incorporate genomic data often turn to complex, computationally intensive “black-box” models like Random Forests or neural networks.

This research bridges that gap by demonstrating that a simple, interpretable MLR model can achieve high predictive accuracy by prioritizing MC data, specifically the abundance of key phyla, over traditional inputs. This approach was validated on a scarcely explored substrate mixture PM and CD, which are significant agro-industrial residues in regions like Valle del Cauca, Colombia. The model’s utility is further enhanced by its synergy with low-cost IoT monitoring systems, which offer a practical solution for real-time data acquisition in decentralized settings. By enabling accurate methane prediction with reasonable computational demand, this work presents an accessible and robust tool for optimizing biogas management in the rural and resource-limited contexts where it is most needed.

Considering the analysis was conducted over a limited timeframe within a single AD process, the outcomes provide a solid foundation for evaluating the predictive relationship between microbial composition and methane concentration. Expanding the evaluation to longer periods and incorporating diverse feedstocks, reactor designs, and operational conditions would support a more comprehensive assessment of the model’s versatility.

In parallel, investigating the metabolic contributions of the identified microorganisms may yield additional insight that enriches the statistical perspective. Integrating the model with real-time monitoring of microbial populations, volatile fatty acid profiles, and key process variables could facilitate adaptive management strategies and enhance the efficiency of AD systems.

## 4. Conclusions

This study successfully developed a MLR model to predict methane concentration in anaerobic codigestion using integrated microbial and operational data. The model demonstrated high predictive accuracy (R^2^ = 0.92, MRE = 6.50%) while requiring only 12 key predictors, substantially reducing complexity compared to the initial 31 variable set. Among the relevant findings, the identification of Verrucomicrobia as a significant predictor was particularly noteworthy, as this phylum is typically associated with extreme environments rather than mesophilic digesters, suggesting previously unrecognized ecological adaptations. The overwhelming dominance of microbial indicators over conventional process parameters highlights the critical importance of community dynamics in driving methanogenic performance. Furthermore, the moving window approach with Ridge regularization effectively captured the system’s biological evolution while maintaining robustness against overfitting. This modeling approach demonstrates significant potential for practical implementation in rural and resource-limited settings, offering a viable method for methane prediction without sophisticated computational requirements.

Future work should focus on validating this model across diverse reactor configurations and feedstock types to assess its generalizability. Additionally, developing cost-effective molecular monitoring tools for the identified key microbial groups could enable real-time implementation of this predictive approach in practical applications.

## Figures and Tables

**Table 1:** Substrate characterization.

Substrate	C (%)	N (%)	C:N	%Hum.	%TS	%VS	%VS/%TS	%FS
I	32.9	3.2	10.3	98.0%	2.2%	1.3%	60.8%	0.9%
CD	43.1	0.9	45.9	11.0%	89.0%	85.4%	96.0%	3.6%
PM	12.9	1.9	7.0	72.0%	28.0%	21.0%	75.0%	7.0%

I: Inoculum; CD: Cassava dregs; PM: Pig manure; C: Carbon content: N: nitrogen content.

**Table 2:** Reactor feeding regime.

D1F1															
Mix	OLR (gVS/L·day)	Mix Load (g)	I (g)	PM (g)	CD (g)	H_2_O Added (g)	Daily Load (g)	%TS	C:N	%I	%PM	%CD	HRT (day)	Period (day)	pH Treatment
I	12.40	329	329			152	481	10%	10.3	100%	0%	0%	5	0–5	
1	7.67	48		35	13	166	214	10%	21.6	0%	73%	27%	11	6–27	Lime
2	7.66	54		43	11	164	218	10%	18.3	0%	80%	20%	11	28–49	
3	6.60	48		39	9	162	210	9%	17.5	0%	81%	19%	11	50–89	NaOH
4	5.90	46	14	25	7	165	211	8%	15.7	30%	54%	15%	11	90–118	NaOH
5	5.87	71	35	26	10	136	207	8%	20.7	50%	36%	14%	11	119–161	
**D1F2**															
I	12.40	439	439			202	641	10%	10.3	100%	0%	0%	5	0–5	
1	5.75	48		35	13	166	214	10%	21.6	0%	73%	27%	15	6–38	
2	5.74	54		43	11	164	218	10%	18.3	0%	80%	20%	15	39–60	
3	4.95	48		39	9	162	210	9%	17.5	0%	81%	19%	15	61–100	
4	4.41	46	14	25	7	165	211	8%	15.7	30%	54%	15%	15	101–127	
5	4.40	71	35	26	10	136	207	8%	20.7	50%	36%	14%	15	128–161	

I = inoculum; PM = pig manure; CD = cassava dreg; OLR = organic loading rate; TS = total solids; HRT = hydraulic retention time.

**Table 3:** Data processing.

D1F1					
Steps	pH	T (°C)	CH_4_	Total	%
All data	694,110	694,110	694,110	2,082,330	100%
Day-hour	694,110	694,110	694,110	2,082,330	100%
Filters	635,953	635,953	635,953	1,907,859	92%
MICE	694,110	694,110	694,110	2,082,330	100%
Data per hour	2893	2893	2893	8679	0.42%
Data per day	152	152	152	456	0.02%
**D1F2**					
All data	573,215	573,215	573,215	1,719,645	100%
Day-hour	573,215	573,215	573,215	1,719,645	100%
Filters	518,897	518,897	518,897	1,556,691	91%
MICE	573,215	573,215	573,215	1,719,645	100%
Data per hour	2389	2389	2389	7167	0.42%
Data per day	147	147	147	441	0.03%

**Table 4:** List of associated suffixes for VFA (a).

Acetic	1	Caproic	5
Propionic	2	Heptanoic	6
Butyric	3	Ethanol	7
Valeric	4	Propanol	8

**Table 5:** List of associated suffixes for microorganisms (m).

Firmicutes	9	Tenericutes	19
Bacteroidetes	10	Armatimonadetes	20
Actinobacteria	11	Cyanobacteria Chloroplast	21
Proteobacteria	12	Acidobacteria	22
Planctomycetes	13	Lentisphaerae	23
Synergistetes	14	BRC1	24
Spirochaetes	15	Candidatus Saccharibacteria	25
Euryarchaeota	16	Parcubacteria	26
Verrucomicrobia	17	Chloroflexi	27
Cloacimonetes	18		

**Table 6:** List of associated suffixes for operating conditions (p).

phi	28	pho	30
Ti	29	To	31

**Table 7:** Approximation coefficients.

$C_{1}$	−0.25	$C_{17}$	152.94
$C_{2}$	0.72	$C_{18}$	−154.01
$C_{3}$	4.86	$C_{19}$	−112.07
$C_{4}$	18.20	$C_{20}$	165.65
$C_{5}$	−61.33	$C_{21}$	60.94
$C_{6}$	−22.60	$C_{22}$	15.47
$C_{7}$	−12.08	$C_{23}$	−302.56
$C_{8}$	14.33	$C_{24}$	159.10
$C_{9}$	7.39	$C_{25}$	−450.57
$C_{10}$	−18.99	$C_{26}$	81.69
$C_{11}$	−21.21	$C_{27}$	116.88
$C_{12}$	−161.69	$C_{28}$	−1562.95
$C_{13}$	−58.79	$C_{29}$	6.72
$C_{14}$	67.72	$C_{30}$	68.50
$C_{15}$	123.17	$C_{31}$	242.54
$C_{16}$	84.58		

**Table 8:** Weighting parameters $C_{i}^{*}$.

$C_{10}^{*}$	66.48	$C_{27}^{*}$	1.79
$C_{12}^{*}$	50.51	$C_{3}^{*}$	1.53
$C_{17}^{*}$	38.48	$C_{4}^{*}$	1.43
$C_{13}^{*}$	36.87	$C_{26}^{*}$	0.88
$C_{15}^{*}$	36.06	$C_{8}^{*}$	0.67
$C_{11}^{*}$	34.35	$C_{6}^{*}$	0.57
$C_{20}^{*}$	30.92	$C_{22}^{*}$	0.37
$C_{14}^{*}$	28.37	$C_{21}^{*}$	0.34
$C_{9}^{*}$	15.61	$C_{1}^{*}$	0.17
$C_{16}^{*}$	14.37	$C_{28}^{*}$	0.15
$C_{19}^{*}$	12.25	$C_{2}^{*}$	0.13
$C_{18}^{*}$	7.87	$C_{31}^{*}$	0.11
$C_{23}^{*}$	7.63	$C_{7}^{*}$	0.11
$C_{25}^{*}$	4.04	$C_{30}^{*}$	0.01
$C_{24}^{*}$	2.30	$C_{29}^{*}$	0.01
$C_{5}^{*}$	2.21		

**Table 9:** New weighted approximation coefficients.

$C_{10}$	−4.64	$C_{20}$	63.94
$C_{12}$	−42.40	$C_{14}$	16.20
$C_{17}$	−94.76	$C_{9}$	1.70
$C_{13}$	−7.66	$C_{16}$	−7.10
$C_{15}$	64.88	$C_{19}$	−22.13
$C_{11}$	−4.02	$C_{18}$	251.39

**Table 10:** Precision metrics for approximations and real data.

	$R^{2}$	MRE [%]	RMSE [ppm]
All variables	0.989	12.59	319.94
Weighted approximation	0.979	14.94	435.82

**Table 11:** Evaluation of the training model and predictive model.

	$R^{2}$	MRE [%]	RMSE [ppm]
Training Fit	0.999	0.35	20.77
Prediction	0.920	6.50	139.84

**Table 12:** Performance comparison of predictive models in anaerobic digestion.

Reference	Model Type	Key Predictors	Performance (R^2^ or Error)	Key Finding/Novelty
[44]	ML Algorithms (RF, NNET, etc.)	Operational and genomic data.	RF Accuracy = 0.82	Combines data types but requires more complex models for slightly lower performance.
[78]	Multilayer Perceptron (MLP)	High-resolution SCADA data.	MLP (SCADA): Adj. $R^{2}$ = 0.78	Relies on massive, high-frequency operational data, concluding that microbial input is unnecessary.
[50]	MLP optimized with metaheuristics.	Operational parameters.	ERWCA-MLP (test): $R^{2}$ = 0.93	Achieves high accuracy but requires highly complex, computationally intensive “black-box” models.
[30]	Temperature-adjusted MLR.	MSW load and air temperature.	Adjusted MLR: $R^{2}$ = 0.975	Also uses a simple MLR but succeeds by excluding microbial data due to its stability in that specific system.
Present Work	Dynamic Multiple Linear Regression (MLR).	12 key microbial phyla.	MLR (test): $R^{2}$ = 0.920	Simple and interpretable model by demonstrating that microbial data can be a critical predictor.

## Data Availability

The original contributions presented in this study are included in the article/Supplementary Material. Further inquiries can be directed to the corresponding author(s).

## Supplementary Materials

The following supporting information can be downloaded at https://www.mdpi.com/article/10.3390/bioengineering12111133/s1, Table S1: Phylum level aggregate counts with no imputed data; Table S2: Cleaned database for initial modeling.
